# Whole blood gene expression moderates associations between AD biomarkers and cognitive decline in cognitively unimpaired older adults

**DOI:** 10.1002/alz.71225

**Published:** 2026-02-22

**Authors:** Hannah M. Klinger, Mabel Seto, Vaibhav A. Janve, Jane A. Brown, Michelle Clifton, Colin Birkenbihl, Gillian T. Coughlan, Diana L. Townsend, Michael Properzi, Jane Zyski, Ting‐Chen Wang, Rebecca E. Amariglio, Kathryn V. Papp, Dorene M. Rentz, Hyun‐Sik Yang, Jasmeer Chhatwal, Michael C. Donohue, Rema Raman, Robert A. Rissman, Paul Aisen, Keith A. Johnson, Reisa A. Sperling, Logan Dumitrescu, Timothy Hohman, Rachel F. Buckley

**Affiliations:** ^1^ Department of Neurology Massachusetts General Hospital and Harvard Medical School Boston Massachusetts USA; ^2^ Center for Alzheimer's Research and Treatment Brigham and Women's Hospital and Harvard Medical School, Boston Boston Massachusetts USA; ^3^ Vanderbilt Memory & Alzheimer's Center Vanderbilt University Medical Center Nashville Tennessee USA; ^4^ Alzheimer's Therapeutic Research Institute University of Southern California San Diego California USA; ^5^ Melbourne School of Psychological Sciences University of Melbourne Melbourne Victoria Australia

**Keywords:** amyloid, longitudinal cognition, PACC, PET, RNAseq, sex differences, tau, whole blood gene expression

## Abstract

**INTRODUCTION:**

Early biological pathways explaining the risk for Alzheimer's disease (AD)–related cognitive decline remain poorly understood.

**METHODS:**

Using linear mixed‐effects models, we investigated whether whole blood gene expression (RNA sequencing) moderates the relationship between AD biomarkers measured by amyloid beta (Aβ) and tau‐PET (positron emission tomography) imaging and longitudinal cognition in 770 cognitively unimpaired older adults (Age_mean _= 71.3, 62% female) from Anti‐Amyloid Treatment in Asymptomatic Alzheimer's (A4) and Longitudinal Evaluation of Amyloid Risk and Neurodegeneration (LEARN) (A4/LEARN).

**RESULTS:**

We identified protective and AD risk–related gene expression signatures on the autosome and X chromosome. Six genes (n_genes_(%); 2(33%) X‐linked) interacted with Aβ‐PET, whereas 103 genes (3(3%) X‐linked) interacted with neocortical tau‐PET, to influence cognitive decline. A total of 110 genes (17(15%) X‐linked) and 3156 genes (121(4%) X‐linked) were moderated by both sex and Aβ‐ or tau‐PET, respectively. Pathway enrichment analyses reflected immunity, protein synthesis, and lipid metabolism.

**DISCUSSION:**

These findings underscore the importance of peripheral transcriptomic markers in identifying sex‐differentiated pathways related to risk of and protection from cognitive decline in preclinical AD.

## Highlights

- Whole blood gene expression moderates biomarker–cognition associations in preclinical AD
- Six genes interacted with amyloid beta positron emission tomography (Aβ‐PET) and 103 with tau‐PET to influence cognitive decline
- Over 3000 gene‐by‐sex interactions reveal sex‐specific transcriptomic vulnerability
- Pathways implicate immunity, ribosomal biology, and vesicle trafficking processes
- Findings support blood‐based, sex‐aware biomarkers for precision AD risk stratification

## 1 BACKGROUND

Alzheimer's disease (AD) is a complex neurodegenerative disorder that unfolds gradually over decades, with pathogenic cascades beginning long before the onset of detectable clinical symptoms. Although post‐mortem brain transcriptomic studies have identified molecular pathways associated with end‐stage AD pathology and cognitive impairment,^1^, ^2^, ^3^, ^4^ less is known about the peripheral biological signals that emerge during the earliest, preclinical phases of the disease.

RNA sequencing (RNAseq) of whole blood offers a scalable and minimally invasive window into systemic biological processes that may interact with AD‐related brain aging and cognitive decline. Early work hints at blood‐based gene expression correlating with key in vivo AD biomarkers such as amyloid beta (Aβ)^5^; yet few studies have examined whether these transcriptomic profiles predict prospective cognitive decline in individuals who are cognitively unimpaired at baseline. Furthermore, mounting evidence highlights the importance of sex as a biological variable in shaping AD risk trajectories,^6^, ^7^, ^8^ with women showing differential vulnerability to Aβ and tau‐moderated cognitive decline.^7^, ^9^ It remains unclear whether blood‐based gene expression signatures capture sex‐specific pathways of vulnerability to or resilience against cognitive decline in the context of AD biomarker burden.

In this study, we leveraged the large, well‐characterized Anti‐Amyloid Treatment in Asymptomatic Alzheimer's (A4)^10^ and Longitudinal Evaluation of Amyloid Risk and Neurodegeneration (LEARN) cohorts to identify peripheral transcriptomic signals associated with longitudinal cognitive trajectories in 770 cognitively unimpaired older adults. Although we have previously published evidence that both Aβ and tau‐PET (positron emission tomography) levels are strong predictors of future cognitive decline in the A4/LEARN cohorts,^11^ there is still considerable unexplained heterogeneity in rates of cognitive decline. By modeling interactions between baseline Aβ and tau‐PET burden, sex, and whole blood gene expression gathered at screening on prospective cognitive decline, we aimed to elucidate early biological pathways that may contribute to individualized risk for cognitive decline in preclinical AD. These findings inform the development of in vivo blood‐based biomarkers, particularly those that are sex aware, to support precision prevention strategies in AD and related dementias (ADRD).

## 2 METHODS

### 2.1 Participants

Data were obtained from individuals enrolled in the A4 clinical trial and the adjoining LEARN observational study.^10^, ^11^ In the A4 study, participants with elevated Aβ‐PET at screening and who met all inclusion and exclusion criteria, were assigned randomly in a 1:1 ratio to receive intravenous solanezumab or placebo. Elevated Aβ‐PET at screening was defined as a standardized uptake value ratio (SUVR) threshold of greater than or equal to 1.10. Participants who were otherwise eligible for A4 but did not show elevated amyloid (SUVR <1.10) were eligible to enroll in the LEARN study and underwent the same cognitive and functional assessments as the A4 study participants.^11^ The A4 study protocol was approved by institutional review boards (IRBs) at each study site, and all participants provided written informed consent. This study followed ethical guidelines stipulated by the Massachusetts General Brigham Human Research Committee. All data were de‐identified and are publicly available at https://www.a4studydata.org/. Data from individuals missing necessary datapoints, did not have whole blood gene expression data, or that did not have at least two timepoints of cognitive assessments, were excluded from the present study, resulting in 770 participants (A4 = 459 [solanezumab = 223 (48.58%), placebo = 236 (51.42%)]; LEARN = 311).

### 2.2 Neuropsychological evaluation

Global cognition was assessed using the four‐component Preclinical Alzheimer's Cognitive Composite (PACC),^12^ a z‐scored and summed composite of four neuropsychological tests: the Free and Cued Selective Reminding Test (FCSRT),^13^ Logical Memory Delayed Recall,^14^, ^15^ the Digit‐Symbol Substitution Test (DSST) from the Wechsler Adult Intelligence Scale‐Revised,^16^ and the Mini‐Mental Status Examination (MMSE).^17^ Three versions of the component subtests were alternated to minimize practice effects, and testing was conducted by trained site psychometrists blinded to treatment arm assignment and adverse events.^11^

### 2.3 Positron emission tomography

Aβ‐PET imaging was acquired for participants using the ^18^F‐Florbetapir (FBP) PET tracer. Scans were acquired 50–70 min post‐injection of the contrast agent. We used a published cortical neocortical composite SUVR referenced to the whole cerebellum.^18^ Pre‐randomization baseline FBP‐PET data were used in our analyses.

Tau‐PET imaging was acquired using the ^18^F‐flortaucipir (FTP) radiotracer. Scans were collected 75–105 min post‐injection, consistent with established acquisition protocols for this tracer.^19^, ^20^ All images were processed using a linear normalization method and semi‐automated pipeline to ensure consistency across sites and scanners, as described in the A4 PET SUVR methods documentation (accessed via www.synapse.org, syn61250768).^21^ Regional SUVRs were computed using a cerebellar gray reference region. Tau‐PET data were not partial volume corrected. For this study, we developed two Tau‐PET regional composites based on region of interest (ROI) variables available in the publicly available dataset to capture baseline medial temporal lobe (MTL) and neocortical (NEO) tau burden. MTL and NEO composites were calculated as the unweighted average of bilateral regional values. The MTL composite included the amygdala, entorhinal cortex, and parahippocampal gyrus. The NEO composite included the fusiform gyrus, middle temporal gyrus, inferior parietal cortex, and inferior temporal gyrus.

**RESEARCH IN CONTEXT**

1. **Systematic review**: We reviewed the published literature in PubMed and preprint archives on blood‐based gene expression, amyloid, and tau‐PET (positron emission tomography) biomarkers, and cognitive decline in Alzheimer's disease (AD). Prior studies have linked peripheral transcriptomic profiles to amyloid burden, but few have examined their interaction with tau or prospective cognition in preclinical populations. Evidence on sex‐specific transcriptomic pathways in AD is especially sparse.
2. **Interpretation**: In 770 cognitively unimpaired older adults from the Anti‐Amyloid Treatment in Asymptomatic Alzheimer's (A4) and Longitudinal Evaluation of Amyloid Risk and Neurodegeneration (LEARN) (A4/LEARN) cohorts, we found that whole blood gene expression moderates associations between amyloid and tau‐PET burden and longitudinal cognitive trajectories. More than 3000 genes demonstrated sex‐specific interactions, revealing transcriptomic vulnerability that diverges between men and women. Pathway analyses implicated immune signaling, ribosomal biology, and vesicle trafficking in shaping early cognitive decline.
3. **Future directions**: Replication in diverse cohorts and mechanistic studies are needed to validate these transcriptomic markers and determine their causal role. Integrating sex‐aware transcriptomic signals into biomarker frameworks could improve early detection and risk stratification, advancing precision prevention strategies for AD.

### 2.4 Open‐label extension period

Participants in the A4 placebo arm were able to proceed to solanezumab treatment during the open‐label extension (OLE) period.^22^ To account for any residual treatment effects during the OLE period, we calculated cumulative treatment dose as the cumulative sum of treatment dose received at each time point and adjusted for this in our analyses.

### 2.5 Blood collection and RNAseq processing

Whole blood samples (2.5 mL) were collected in PAXgene tubes, frozen on site, shipped on dry ice, and stored at −80°C. All samples were processed and sequenced by the VANTAGE sequencing core (https://www.vumc.org/vantage/home) at Vanderbilt University Medical Center (Nashville, TN, USA). Total RNA was extracted from whole blood using the QIASymphony RNA Kit (QIAGEN, 931636), and both ribosomal RNA and hemoglobin were depleted with the NEBNext Globin and rRNA Depletion Kit (New England BioLabs, Inc., E7750). Library preparation was completed using the NEBNext Ultra Directional Library Prep Kit (New England BioLabs, Inc., E7420) before sequencing was performed using 151 base pair (bp) paired end reads on an Illumina NovaSeq 6000 (Illumina), targeting an average of 60 million reads per sample. Quality control was performed by the Vanderbilt Memory and Alzheimer's Center following bulk RNAseq pipelines described by the Accelerating Medicines Partnership ‐ Alzheimer's Disease Target Discovery and Preclinical Validation program (AMP‐AD).^23^, ^24^ Gene‐level counts were quantile normalized across samples and adjusted for technical variation (e.g., batch), consistent with AMP‐AD bulk RNAseq pipelines and as previously described.^24^

### 2.6 Statistical analyses

All analyses were run in R version 4.4.0. A series of linear mixed‐effects models (detailed below) were used to examine the relationship between both autosomal and X‐linked gene expression and longitudinal cognitive decline. We also explored the moderating effects of sex and AD biomarkers, including continuous Aβ‐PET and MTL and NEO tau‐PET burden, on gene expression and cognitive trajectories. All models adjusted for baseline age × Time, years of education × Time, cohort (A4 Treated vs A4 Placebo vs LEARN) × Time, PACC version, and cumulative dose. PACC version and cumulative dose are time‐varying covariates and are not interacted with time in the models. All analyses were corrected for multiple comparisons using a false discovery rate (FDR) corrected *p* < 0.05. The primary models were:

1. Longitudinal PACC ∼ Gene Expression × Time + Covariates
2. Longitudinal PACC ∼ Gene Expression × Sex × Time + Covariates
3. Longitudinal PACC ∼ Gene Expression × AD Biomarker × Time + Covariates
4. Longitudinal PACC ∼ Gene Expression × Sex × AD Biomarker × Time + Covariates

Sex‐stratified models were used to further assess genes that significantly interacted with sex. Sensitivity analyses additionally covaried for baseline Aβ‐PET or were adjusted for each individual's baseline PACC scores, where relevant. Briefly, baseline PACC scores were subtracted from all PACC scores, setting the intercept to 0.

For visualization purposes only, we adapted the primary endpoint analysis used in the A4 trial,^25^ which used a spline basis expansion of time (natural cubic splines with 2 degrees of freedom) to model change in PACC. Post hoc gene set enrichment analyses were performed using the *fgsea* R package (version 1.32.0),^26^ based on pre‐ranked beta estimates from each model. Gene Ontology: Biological Process terms were used for pathway annotation.^27^, ^28^

## 3 RESULTS

A4 and LEARN participants were on average 71.3 (4.7) years old, 62.3% female, and had over 16 years of education (Table 1). Participants completed an average of 12 timepoints of PACC data across an average of 5.3 (2) years of follow‐up, including the OLE period.

**TABLE 1:** Participant demographics by cohort and sex.

	Overall	A4 (Aβ+) *N* = 459		LEARN (Aβ–) *N* = 311		
Characteristic	*N* = 770^a^	Female *N* = 291^a^	Male *N* = 168^a^	Female *N* = 189^a^	Male *N* = 122^a^	*p*‐value^c^, ^d^
Baseline age	71.32 (4.68) [65.00, 85.51]	71.43 (4.60) [65.00, 85.51]	72.90 (5.04) [65.00, 85.26]	70.28 (4.17) [65.00, 84.46]	70.49 (4.53) [65.03, 84.30]	**<0.001**
Years of education	16.57 (2.62) [7.00, 30.00]	16.12 (2.50) [7.00, 22.00]	17.33 (2.91) [10.00, 30.00]	16.19 (2.47) [9.00, 27.00]	17.16 (2.34) [12.00, 26.00]	>0.9
*APOE ε*4 status [ε4+]	343 (44.66%)	165 (56.70%)	101 (60.12%)	43 (22.75%)	34 (28.33%)	**<0.001**
Baseline PACC score	−0.97 (2.36) [−11.15, 5.17]	−0.75 (2.41) [−11.15, 5.17]	−1.84 (2.31) [−7.71, 3.75]	−0.39 (2.29) [−6.79, 4.38]	−1.19 (2.06) [‐5.96, 3.50]	**0.013**
Baseline global Aβ‐PET burden	1.19 (0.21) [0.79, 2.00]	1.33 (0.17) [1.01, 2.00]	1.32 (0.16) [1.01, 1.75]	1.00 (0.07) [0.84, 1.15]	0.99 (0.07) [0.79, 1.15]	**<0.001**
PACC timepoints	12.08 (4.11) [2.00, 18.00]	12.38 (4.05) [2.00, 18.00]	12.18 (4.13) [2.00, 18.00]	11.96 (4.13) [2.00, 18.00]	11.43 (4.14) [2.00, 18.00]	**0.041**
PACC years of follow‐up	5.27 (2.04) [0.12, 7.60]	5.29 (1.94) [0.18, 7.46]	5.18 (2.00) [0.22, 7.46]	5.41 (2.15) [0.12, 7.60]	5.15 (2.17) [0.29, 7.53]	**0.011**
Randomized treatment group [solanezumab group]	223 (48.58%)	134 (60.09%)	89 (39.99%)	NA^b^	NA^b^	–

^a^ Mean (SD) [Range], n (%).

^b^ LEARN participants are not randomized into treatment groups.

^c^ Wilcoxon rank‐sum test; Pearson's chi‐square test.

^d^ Group comparisons across A4 and LEARN (not stratified by sex); PACC, Preclinical Alzheimer's Cognitive Composite.

### 3.1 Gene expression on cognitive decline

There were no significant gene associations with longitudinal cognition after adjusting for covariates and FDR correction (Table S1).

### 3.2 Sex by gene expression on cognitive decline

We found no sex by gene interactions on longitudinal cognition after adjusting for covariates and FDR correction (Table S2).

### 3.3 Aβ‐PET by gene expression on cognitive decline

**FIGURE 1:**
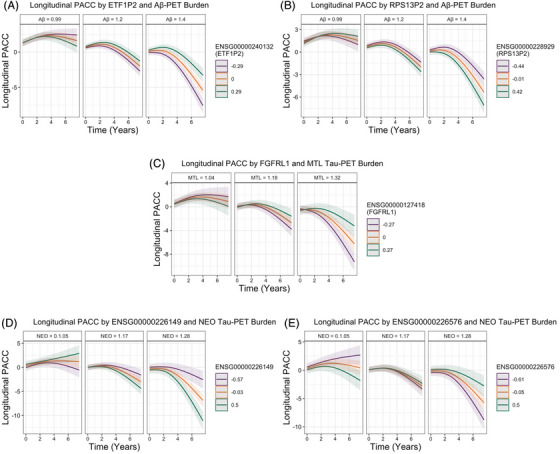
Longitudinal Preclinical Alzheimer's Cognitive Composite (PACC) trajectories by **A**. Baseline Aβ‐PET burden and *ETF1P2* expression, **B**. Baseline Aβ‐PET burden and *RPS13P2* expression, **C**. Baseline medial temporal lobe (MTL) tau‐PET burden and *FGFRL1* expression, **D**. Baseline neocortical (NEO) tau‐PET burden and ENSG00000226149 expression, and **E**. Baseline NEO tau‐PET burden and ENSG00000226576 expression. For visualization purposes, we adapted the primary endpoint analysis used in the A4 trial, which used a spline basis expansion of time (natural cubic splines with 2 degrees of freedom) to model change in PACC.

Six genes moderated the association between Aβ‐PET and cognitive decline (Table 2). Two X‐linked and four autosomal genes were identified. Lower expression of *ETF1P2* (chr13; Figure 1A), *MIRLET7F2* (X‐linked), *RHOXF2* (X‐linked), and *SMIM18* (chr13) and higher baseline Aβ‐PET burden were associated with faster rates of cognitive decline. Alternatively, greater expression of *RPS13P2* (chr1; Figure 1B) and *ORC2* (chr2) and higher baseline Aβ‐PET burden were associated with faster cognitive decline (i.e., greater risk). All gene associations are shown in Table S3.

**TABLE 2:** Standardized model estimates for gene × Aβ‐PET × time interaction.

Ensembl gene ID	Gene name	Chromosome name	Standardized beta	Standard error	*t*‐value	*p*‐value	FDR *p*‐value
ENSG00000240132	*ETF1P2*	7	0.084	0.017	5.061	<0.001	0.009
ENSG00000228929	*RPS13P2*	1	−0.077	0.017	−4.664	<0.001	0.018
ENSG00000208012	*MIRLET7F2*	X	0.072	0.015	4.654	<0.001	0.018
ENSG00000115942	*ORC2*	2	−0.079	0.017	−4.648	<0.001	0.018
ENSG00000131721	*RHOXF2*	X	0.077	0.017	4.579	<0.001	0.020
ENSG00000253457	*SMIM18*	8	0.072	0.016	4.478	<0.001	0.026

Table is ordered by descending effect size.

### 3.4 Tau‐PET by gene expression on cognitive decline

One autosomal gene moderated the association between MTL tau and cognitive decline; lower expression of *FGFRL1* (chr4) was associated with faster cognitive decline in participants with high baseline MTL tau‐PET burden (Figure 1C). All MTL tau‐PET gene associations are shown in Table S4.

We found that 103 genes (three X‐linked) moderated the association between NEO tau and cognitive decline. All gene associations are shown in Table S5. Full Ensembl IDs are given for genes that have no other known identifier. The top 10 genes with the greatest effect sizes (Table 3) included genes with large negative moderating associations with cognitive decline, such as ENSG00000226149 (chr6; Figure 1D), *PSENEN* (chr19), ENSG00000279742 (chr11), *PPP4C* (chr16), ENSG00000289419 (chr1), and ENSG00000272109 (chr5). That is, lower expression and higher baseline NEO tau was associated with faster rates of cognitive decline. Positively moderated genes included ENSG00000226576 (chr10; Figure 1E), ENSG00000236998 (chr9), *MYB* (chr6), and *MMGT1* (X‐linked); higher expression coupled with higher NEO tau was associated with faster cognitive decline. Overall, 79 genes (76%), including those highlighted, remained significant when additionally covarying for baseline Aβ‐PET in sensitivity analyses (Table S6).

**TABLE 3:** Select standardized model estimates for gene × NEO tau‐PET × time interaction.

Ensembl Gene ID	Gene name	Chromosome name	Standardized beta	Standard Error	*t*‐value	*p*‐value	FDR *p*‐value
ENSG00000226149	NA^a^	6	−0.186	0.034	−5.406	<0.001	0.001
ENSG00000226576	NA^a^	10	0.156	0.031	5.004	<0.001	0.006
ENSG00000205155	*PSENEN*	19	−0.130	0.026	−4.902	<0.001	0.006
ENSG00000279742	NA^a^	11	−0.127	0.026	−4.862	<0.001	0.006
ENSG00000149923	*PPP4C*	16	−0.146	0.031	−4.686	<0.001	0.010
ENSG00000289419	NA^a^	1	−0.139	0.030	−4.679	<0.001	0.010
ENSG00000236998	NA^a^	9	0.144	0.031	4.585	<0.001	0.012
ENSG00000272109	NA^a^	5	−0.138	0.030	−4.573	<0.001	0.012
ENSG00000118513	*MYB*	6	0.147	0.032	4.554	<0.001	0.012
ENSG00000169446	*MMGT1*	X	0.154	0.034	4.539	<0.001	0.012

^a^ No corresponding gene name. Table is ordered by descending effect size.

### 3.5 Aβ‐PET by sex by gene expression on cognitive decline

**FIGURE 2:**
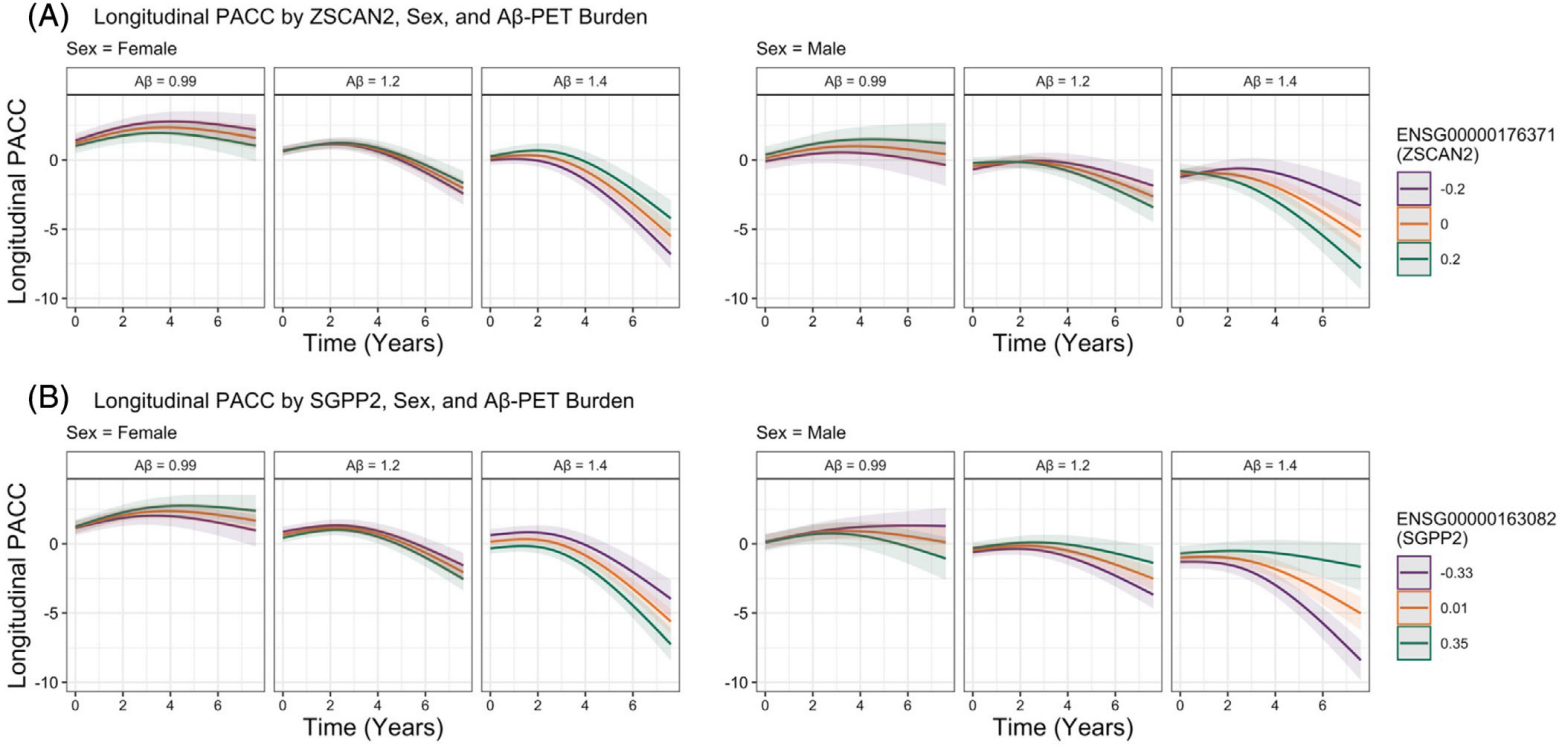
Longitudinal Preclinical Alzheimer's Cognitive Composite (PACC) trajectories by sex, baseline Aβ‐PET burden, and (**A)** *ZSCAN2* expression and (**B)** *SGPP2* expression. For visualization purposes, we adapted the primary endpoint analysis used in the A4 trial, which used a spline basis expansion of time (natural cubic splines with 2 degrees of freedom) to model change in PACC.

A total of 110 genes (17 X‐linked) were moderated by an Aβ‐PET × sex interaction after adjusting for covariates and FDR correction (Table S7). Of the top 10 genes with the greatest effect size (Table 4), greater *ZSCAN2* (chr15; Figure 2A), *GDPD2 *(X‐linked), *FAM223A* (X‐linked), *UBXN10* (chr1), *UBE2D3P2* (chr3), and *KLHL4 *(X‐linked) were associated with faster cognitive decline among males with higher baseline Aβ‐PET burden relative to females. Alternatively, lower *SGPP2* (chr14; Figure 2B), *SUV39H2‐DT* (chr10), *DNPH1* (chr6), and ENSG00000287729 (X‐linked; novel transcript) were associated with faster cognitive decline among males with higher Aβ‐PET burden compared to females.

**TABLE 4:** Select standardized model estimates for gene × sex × Aβ‐PET × time interaction.

Ensembl gene ID	Gene name	Chromosome name	Standardized beta	Standard error	*t*‐value	*p*‐value	FDR *p*‐value
ENSG00000176371	*ZSCAN2*	15	−0.172	0.033	−5.159	<0.001	0.005
ENSG00000163082	*SGPP2*	2	0.169	0.034	4.943	<0.001	0.006
ENSG00000130055	*GDPD2*	X	−0.378	0.078	−4.855	<0.001	0.006
ENSG00000279245	*FAM223A*	X	−0.279	0.058	−4.814	<0.001	0.006
ENSG00000162543	*UBXN10*	1	−0.145	0.030	−4.798	<0.001	0.006
ENSG00000272853	*SUV39H2‐DT*	10	0.162	0.034	4.790	<0.001	0.006
ENSG00000112667	*DNPH1*	6	0.171	0.037	4.646	<0.001	0.010
ENSG00000225558	*UBE2D3P2*	3	−0.149	0.033	−4.583	<0.001	0.010
ENSG00000102271	*KLHL4*	X	−‐0.333	0.073	−4.577	<0.001	0.010
ENSG00000287729	NA^a^	X	0.162	0.036	4.562	<0.001	0.010

^a^ No corresponding gene name. Table is ordered by descending effect size.

To better decipher these three‐way interactions, we conducted sex‐stratified analyses. Of the 110 significant genes implicated in cognitive decline through Aβ‐PET and sex, 41% of these genes were significant in both sexes, whereas 13% were significant in only females and 43% were significant in only males (Table S8). In males, we found that greater expression of *ZSCAN2*, *GDPD2 *(X‐linked), *FAM223A* (X‐linked), and *UBXN10* and lower expression of *SGPP2* were associated with faster cognitive decline in those with higher baseline Aβ‐PET. In contrast, greater expression of *SGPP2* and lower expression of *ZSCAN2*, *GDPD2 *(X‐linked), *FAM223A* (X‐linked), and *UBXN10* were associated with faster cognitive decline in females with higher baseline Aβ‐PET.

### 3.6 Tau‐PET by sex by gene expression on cognitive decline

**FIGURE 3:**
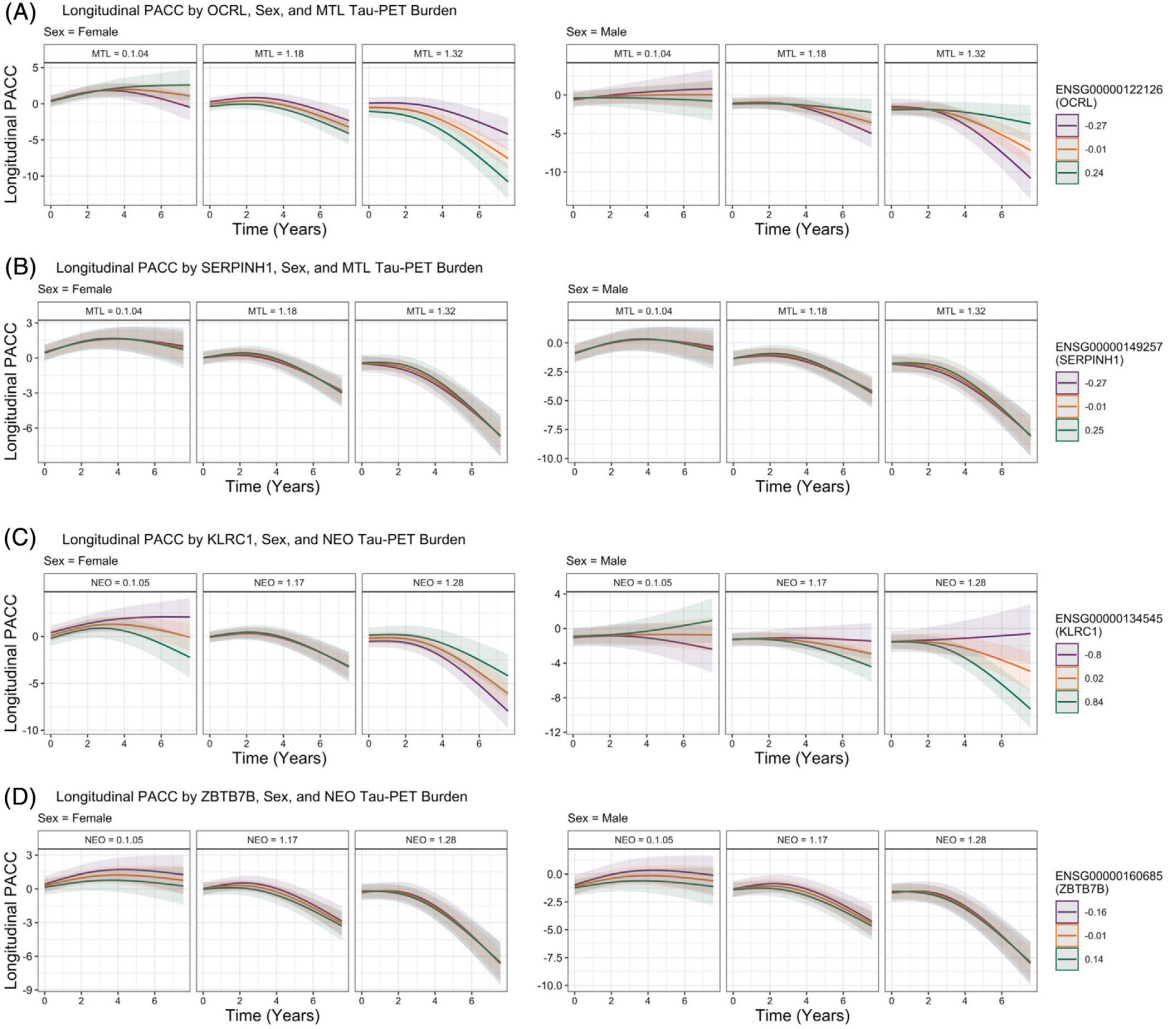
Longitudinal Preclinical Alzheimer's Cognitive Composite (PACC) trajectories by sex and (**A)** baseline MTL tau‐PET burden and *OCRL* expression and (**B)** baseline MTL tau‐PET and *SERPINH1* expression, (**C)** baseline NEO tau‐PET burden and *KLRC1* expression, and (**D)** baseline NEO tau‐PET burden and *ZBTB7B* expression. For visualization purposes, we adapted the primary endpoint analysis used in the A4 trial, which used a spline basis expansion of time (natural cubic splines with 2 degrees of freedom) to model change in PACC.

A total of 112 genes (6 X‐linked) were moderated by a MTL tau‐PET × sex interaction (Table S9). Of the 10 genes with the greatest effect size (Table 5), greater *SERPINH1* (chr11; Figure 3A), ENSG00000284773 (chr1), *PCID2* (chr13), *UBA5* (chr3), *CEP250* (chr20), and *CD226* (chr18) expression were associated with faster cognitive decline in males with higher baseline MTL tau‐PET burden relative to females. Lower *OCRL* (X‐linked; Figure 3B), *ZBTB7B* (chr1), *MTM1* (X‐linked), and *BTBD3* (chr20) expression was associated with faster cognitive decline in males with higher baseline MTL tau‐PET burden relative to females.

**TABLE 5:** Select standardized model estimates for gene × sex × MTL tau‐PET × time interaction.

Ensembl gene ID	Gene name	Chromosome name	Standardized beta	Standard error	*t*‐value	*p*‐value	FDR *p*‐value
ENSG00000122126	*OCRL*	X	0.288	0.058	4.944	<0.001	0.017
ENSG00000149257	*SERPINH1*	11	−0.245	0.052	−4.739	<0.001	0.018
ENSG00000284773	*NA* ^a^	1	−0.320	0.069	−4.642	<0.001	0.018
ENSG00000126226	*PCID2*	13	−0.284	0.062	−4.609	<0.001	0.018
ENSG00000081307	*UBA5*	3	−0.275	0.060	−4.597	<0.001	0.018
ENSG00000160685	*ZBTB7B*	1	0.280	0.063	4.431	<0.001	0.023
ENSG00000171100	*MTM1*	X	0.295	0.067	4.391	<0.001	0.023
ENSG00000126001	*CEP250*	20	−0.257	0.059	−4.369	<0.001	0.023
ENSG00000132640	*BTBD3*	20	0.253	0.058	4.358	<0.001	0.023
ENSG00000150637	*CD226*	18	−0.251	0.058	−4.357	<0.001	0.023

^a^ No corresponding gene name. Table is ordered by descending effect size. MTL, medial temporal lobe.

A total of 3156 genes (121 X‐linked) were moderated by a NEO tau‐PET × sex interaction (Tables S10). Of the top 10 genes with the greatest effect size in the NEO models (Table 6), greater *KLRC1* (chr12; Figure 3C), *TAS2R46* (chr12), *ZBTB44* (chr11), *ZNF892* (chr2), *PCYT2* (chr17), and *PCNT* (chr21) expression were associated with faster cognitive decline among males with higher baseline NEO tau‐PET burden relative to females. Alternatively, lower *ZBTB7B* (chr1; Figure 3D), *NIPA2* (chr15), *KBTBD11* (chr8), and *ALCAM* (chr3) expression in males with higher baseline NEO tau‐PET burden were associated with faster cognitive decline relative to females. A total of 1265 (40%) of the original 3156 genes remained significant when covarying for baseline Aβ‐PET (Table S11).

**TABLE 6:** Select standardized model estimates for gene × sex × NEO tau‐PET × time interaction.

Ensembl gene ID	Gene name	Chromosome name	Standardized beta	Standard error	*t*‐value	*p*‐value	FDR *p*‐value
ENSG00000134545	*KLRC1*	12	−0.319	0.055	−5.805	<0.001	<0.001
ENSG00000226761	*TAS2R46*	12	−0.377	0.065	−5.794	<0.001	<0.001
ENSG00000160685	*ZBTB7B*	1	0.334	0.058	5.775	<0.001	<0.001
ENSG00000196323	*ZBTB44*	11	−0.337	0.058	−5.766	<0.001	<0.001
ENSG00000233757	*ZNF892*	2	−0.347	0.061	−5.734	<0.001	<0.001
ENSG00000140157	*NIPA2*	15	0.350	0.062	5.614	<0.001	<0.001
ENSG00000185813	*PCYT2*	17	−0.324	0.058	−5.614	<0.001	<0.001
ENSG00000176595	*KBTBD11*	8	0.384	0.068	5.603	<0.001	<0.001
ENSG00000160299	*PCNT*	21	−0.336	0.060	−5.558	<0.001	<0.001
ENSG00000170017	*ALCAM*	3	0.378	0.070	5.435	<0.001	<0.001

Table is ordered by descending effect size. NEO, Neocortical.

In addition, we found that 97 genes showed significant effects (i.e., overlap) across both MTL × sex and NEO × sex models (Table S12), suggesting a core set of peripheral transcriptomic signals linked to tau‐related cognitive decline across both the medial temporal and neocortical regions.

We then examined sex‐stratified analyses on the significant genes implicated in cognitive decline through MTL tau‐PET or NEO tau‐PET and sex. Eighty‐one percent of gene by MTL tau‐PET associations were significant in both sexes, 12% were significant in only females, and 7% were significant in only males (Table S13). Of the top five genes, we found that greater *OCRL* and lower *SERPINH1*, ENSG00000284773, *PCID2*, and *UBA5* were associated with faster cognitive decline in males with higher baseline MTL tau‐PET relative to females. By contrast, of the 3156 significant genes implicated in cognitive decline through NEO tau‐PET and sex, 8% of these genes were significant in both sexes, 5% were significant in only females and 83% were significant in only males (Table S14). Of the top five genes, we found that greater *KLRC1*, *TAS2R46, ZBTB44*, and *ZNF892* and lower *ZBTB7B* were associated with faster cognitive decline in males with greater baseline NEO tau‐PET relative to females.

### 3.7 Sensitivity analyses adjusting for baseline cognition

Because baseline PACC is significantly associated with Aβ‐PET and tau‐PET burden in our data (Table S15), we performed sensitivity analyses additionally adjusting for baseline PACC scores in our models. The results of the Aβ‐PET sensitivity analyses remained largely concordant with our previous findings: 6 of 6 genes remained significant and consistent (i.e., same beta direction) in the Aβ‐PET models, and 77 (70%) of 110 genes in the Aβ‐PET × sex interaction models. A smaller proportion of genes remained significant after adjusting for baseline PACC scores in tau‐PET models: 0 of 1 in the MTL tau‐PET model, 59 (57%) of 103 genes in the NEO tau‐PET model, and 77 (69%) of 112 and 1751 (55%) of 3156 genes in MTL and NEO tau‐PET × sex interaction models, respectively (Tables S16–S21).

### 3.8 Pathway enrichment analyses

**FIGURE 4:**
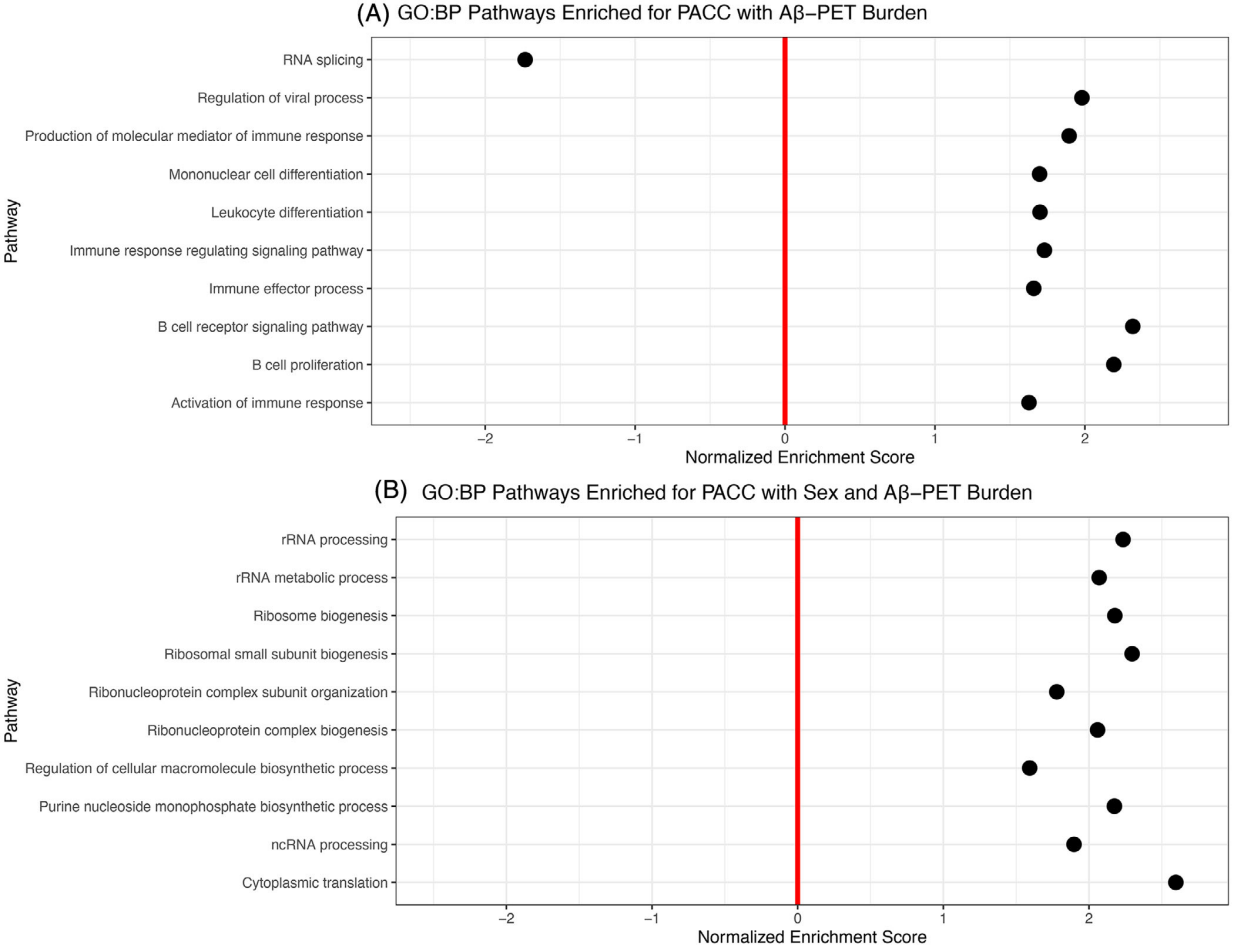
Top 10 most significant biological pathways enriched for PACC by (**A)** Aβ‐PET and (**B)** Aβ‐PET and sex.

**FIGURE 5:**
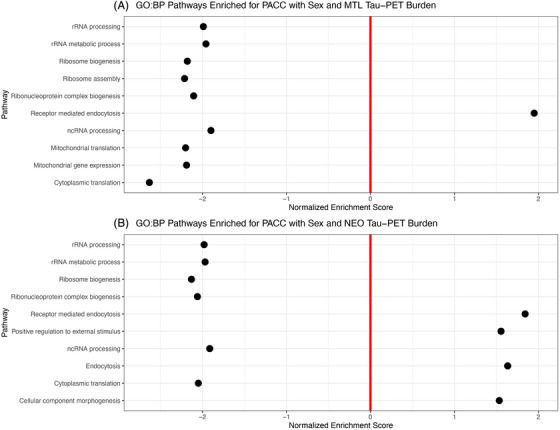
Top 10 most significant biological pathways enriched for PACC by (**A)** MTL tau‐PET and sex and (**B)** NEO tau‐PET and sex.

To link our individual gene results to broader biological processes (defined by gene sets organized by Gene Ontology Database),^27^, ^28^ we performed pathway enrichment analyses using *fgsea*.^26^ These pathway/gene set analyses revealed that 116 biological pathways were enriched for genes associated with cognitive decline by Aβ‐PET (Table S22). The most significantly enriched pathways were predominantly immune related, including activation of the immune response, B cell receptor signaling, leukocyte differentiation, and mononuclear cell differentiation (Figure 4A). Only one pathway—RNA splicing—was negatively associated with cognitive decline. We also found that 27 biological pathways were enriched for cognitive decline by Aβ‐PET and sex (Table S23). These pathways were largely associated with ribosome biology and protein synthesis, including ribosome biogenesis, cytoplasmic translation, RNA processing, and macromolecule biosynthetic processes (Figure 4B). For tau‐PET, 185 biological pathways enriched for cognitive decline by MTL tau‐PET and sex (Table S24). The top pathways again implicated ribosomal and mitochondrial function (e.g., ribosome biogenesis, mitochondrial translation), but were negatively associated with cognitive outcomes, with only receptor‐mediated endocytosis showing a positive enrichment (Figure 5A). Only one pathway was enriched for cognitive decline by NEO tau‐PET (Table S25), but 158 biological pathways were enriched for cognitive decline by NEO tau‐PET and sex (Table S26). These pathways included a mix of negatively enriched translation‐related processes (e.g., RNA processing, cytoplasmic translation) and positively enriched processes involved in vesicle trafficking and external stimulus response (e.g., endocytosis, receptor‐mediated endocytosis, and cellular morphogenesis; Figure 5B).

## 4 DISCUSSION

In this study, we examined the relationship between whole blood gene expression and longitudinal cognition in cognitively unimpaired individuals enrolled in the A4 and LEARN studies. Although no direct effects were observed, we identified 6 genes that were modified by Aβ‐PET, 1 gene moderated by MTL tau‐PET, and 103 genes moderated by NEO tau‐PET on PACC. We also found that many associations were further moderated by sex, with 110 genes moderated by Aβ‐PET and sex, 112 genes moderated by MTL tau‐PET and sex, and 3156 genes moderated by NEO tau‐PET and sex. It is possible that the large number of gene‐moderating findings with NEO tau‐PET and sex may be due to the proximal association between neocortical tau burden and cognitive decline. Moreover, a large proportion of genes moderating NEO tau‐PET remained significant when adjusting for baseline Aβ‐PET, suggesting that there are effects independent of Aβ. Over half of these identified genes (≈56%) also remain significant when adjusting for baseline PACC scores; our results are not solely due to baseline differences in cognition.

Further examination of the genes implicated in cognitive decline when interacted with Aβ‐PET showed that two genes were associated with increased risk and four were protective among individuals with elevated baseline Aβ‐PET. Higher expression of *MIRLET7F2* ^29^ was associated with slower cognitive decline among individuals with elevated Aβ‐PET; it belongs to the let‐7 family of (non‐coding) microRNAs, which regulate multiple aspects of cellular function, including inflammation and neuroprotection, both of which have been linked to AD. let‐7 family members have been observed in the cerebrospinal fluid of patients with AD and other neurodegenerative diseases, and have also been shown to increase in aging tissues.^30^, ^31^

Among individuals with higher baseline Aβ‐PET levels, *ETF1P2* was associated with faster cognitive decline. By contrast, *RPS13P2* was protective, and was associated with slower cognitive decline. *ETF1P2* is a pseudogene related to eukaryotic translation termination factor 1 (ETF1),^32^, ^33^ which is potentially implicated in dysregulation of protein synthesis in neurodegenerative processes. *RPS13P2* is a pseudogene related to ribosomal protein S13,^32^, ^34^ suggesting potential dysregulation of ribosomal function and protein synthesis. Pseudogenes are DNA sequences that share high homology with genes, although they are not protein coding themselves. Emerging evidence suggests a role for pseudogenes in the pathogenesis of AD,^35^ with protein translation implicated in the disease process, although neither *RPS13P2* nor *ETF1P2* has been associated previously with AD. These analyses also identified *SMIM18*,^32^ a small integral membrane protein with limited characterization but potential relevance to cellular homeostasis and *ORC2*, which encodes a subunit of the origin recognition complex (ORC),^32^, ^36^ essential for DNA replication initiation. Together, these findings highlight the potential importance of non‐coding RNA, pseudogenes , and lesser‐known genomic regulators in AD‐related cognitive decline, underscoring the need for further functional investigation into these loci and their roles in neurodegenerative pathways.

Gene set enrichment analyses provided additional support for these findings, with immune‐related pathways (e.g., B cell activation, leukocyte differentiation) enriched among genes moderated by Aβ‐PET burden, consistent with mounting evidence implicating peripheral immune signaling in early AD‐related cognitive decline.^37^, ^38^, ^39^ By contrast, genes moderated by sex and AD biomarkers were enriched for processes related to ribosomal function and protein synthesis. Particularly, transcriptomic vulnerability to tau‐mediated cognitive decline showed sex‐specific enrichment of translation‐related pathways and receptor‐mediated endocytosis and vesicle trafficking. Taken together, these findings suggest that peripheral dysregulation of immune signaling and cellular translation processes may interact with AD biomarkers and sex to influence cognitive decline as early as in the preclinical stage.

Women are disproportionately affected by AD, and they experience faster cognitive decline for a given amount of tau burden relative to men.^7^, ^8^, ^40^ We identified genes that were moderated by sex, suggesting potential biological mechanisms differentiating the sexes to exacerbate or protect from the disease process. It is notable that sex‐stratified analyses implicated gene pathways that were salient for females and males, highlighting the importance of not just focusing on females when considering sex‐specific disease pathways. Notably, we found that more than 3000 genes by neocortical tau interactions were moderated by sex to influence cognitive decline, with 83% found to be male specific. These findings reveal a potentially underappreciated male‐specific transcriptomic vulnerability to tau‐mediated decline, which may be overlooked when focusing on the preponderance of evidence highlighting female‐specific associations with tau burden in post‐mortem and in vivo observational studies.

We found 97 genes that overlapped in their moderation by both MTL and NEO tau on cognitive decline. This select group of genes, given their high bar to reach significance threshold across two sets of anatomically distinct regions of the brain, perhaps promotes them as a high‐value candidate list for further exploration and validation. They may be worthwhile to pursue for downstream functional studies, particularly as these regions reflect different stages of AD‐related tau spread.

Although our study is one of the largest transcriptomic studies of longitudinal cognition in a preclinical AD population, it is not without limitations. First, our data are derived from whole blood; its relevance to the brain has some limitations. Some studies have presented evidence of a connection between peripheral inflammation, for example, and neuroinflammation^41^; however, some of our findings are not inflammatory and therefore require replication in brain tissue or other validation approaches. Many of the hits in this study have known or suspected roles in cell signaling, immune trafficking, lipid metabolism, or autophagy—processes that can bridge across peripheral and central nervous system. As such, not all hope is lost when considering the peripheral system in relation to central nervous system (CNS)–derived disease vulnerability mechanisms. Second, the A4 study is enriched for primarily non‐Hispanic White individuals with elevated amyloid, thus making this sample less generalizable to a broader and more diverse population.

In conclusion, whole blood transcriptomic signals were primarily associated with cognitive decline via interactions with Aβ‐PET, MTL tau‐PET, NEO tau‐PET, Aβ‐PET by sex, MTL tau‐PET by sex, and NEO tau‐PET by sex. The identified genes are implicated in biological processes such as transcriptional regulation, protein synthesis, lipid metabolism, histone methylation, inflammation, and many others. Further work, including validation in external cohorts and mechanistic follow‐up studies, will be critical to elucidate the causal relevance and translational potential of these peripheral transcriptomic markers for early AD risk stratification. Our findings within a preclinical sample may help to inform the development of minimally invasive, in vivo blood‐based biomarkers that could impact AD risk detection and diagnosis (i.e., via blood draws and screening) that may allow for earlier intervention.

## CONFLICT OF INTEREST STATEMENT

H.M.K., M.S., and R.F.B. have no disclosures relevant to this manuscript. R.A.S. has served as a consultant for AbbVie, AC Immune, Acumen, Alector, Apellis, Biohaven, Bristol Myers Squibb, Genentech, Ionis, Janssen, Oligomerix, Prothena, Roche, and Vaxxinity over the past 3 years. She has received research funding from Eisai and Eli Lilly for public–private partnership clinical trials and receives research grant funding from the National Institute on Aging/National Institutes of Health, GHR Foundation, and the Alzheimer's Association. Her spouse, K. Johnson, reports consulting fees from Novartis, Merck, and Janssen. No other authors report disclosures relevant to this manuscript. Author disclosures are available in the supporting information.

## CONSENT STATEMENT

The A4 study protocol was approved by institutional review boards (IRBs) at each study site, and all participants provided written informed consent. This study followed ethical guidelines stipulated by the Massachusetts General Brigham Human Research Committee. All data were de‐identified and are publicly available at https://www.a4studydata.org/.

## Supporting information



Supporting Information

Supporting Information
